# Supplementary material for: Are carbon emissions trading and green financial instruments synergistic? -Comprehensive quantitative research based on content analysis
Source: PLoS One. 2024 Mar 7;19(3):e0298601. doi: 10.1371/journal.pone.0298601 (PMC10919601; doi:10.1371/journal.pone.0298601)
Supplement: S1 Data — (ZIP) [file pone.0298601.s001.zip › Data/地级市Z_F工作报告.zip!/║╙─╧/╜╣╫≈╩╨/└┤╫╘╣1⁄2╓┌║┼í╛┬φ┐╦╩2╛▌═°í┐_2018.docx]

各位代表：

现在，我代表市人民政府，向大会报告工作，请予审议，并请各位政协委员和列席人士提出意见。

一、工作回顾

2014年以来，面对复杂的国际国内形势和繁重的改革发展任务，在省委、省政府和市委的坚强领导下，全市上下以习近平新时代中国特色社会主义思想为指导，坚持稳中求进工作总基调，以新发展理念为引领，以提高发展质量和效益为中心，以推进供给侧结构性改革为主线，着力发挥优势打好“四张牌”，全力克难攻坚、加快转型发展，围绕建设“四个焦作”、早日跻身全省“第一方阵”，建设全面体现新发展理念的示范城市，深入开展“质量、效率、法治”落实年活动，开拓创新、砥砺前行，经济社会保持稳中有进、稳中向好的发展态势。

——综合实力显著增强。2017年，地区生产总值达2342.8亿元，年均增长8.3%，高于全国平均水平1.4个百分点。规模以上工业增加值年均增长9.4%，工业经济跻身全省“第一方阵”。

——结构调整深入推进。二三产业比重达94.1%，较2013年提高1.7个百分点。高新技术产业增加值年均增长15.1%，高于规模以上工业增加值年均增速5.7个百分点。服务业占比较2013年提高5个百分点，年均增长9.4%，高于地区生产总值年均增速1.1个百分点。

——质量效益持续提升。一般公共预算收入达133.8亿元，年均增长8.3%。万元生产总值能耗较2013年下降22.8%，六大高耗能行业增加值占规模以上工业比重较2013年降低4.4个百分点。规模以上工业企业利润达383.2亿元，较2013年增长20.8%。常住人口城镇化率达58%，较2013年提高6个百分点。

——人民生活明显改善。财政民生领域支出累计达657.9亿元，年均增长8.6%。城镇、农村居民人均可支配收入分别达2.92万元、1.62万元，年均增长7.8%、9%。就业、教育、医疗、社保等公共服务水平全面提升，生态环境持续改善，社会大局和谐稳定。总体来看，全市经济社会发展迈上了新的台阶，较好完成了市十二届人大确定的目标任务。

主要做了以下工作：

（一）抓关键稳运行，经济保持平稳增长。主动适应、把握、引领经济发展新常态，抓住重点关键，精准精确施策，经济实现平稳健康发展。一是加强运行调控。出台支持工业发展28条意见、支持制造业高质量发展10条意见、城市建设“1+N”政策体系、加快金融产业发展意见等政策措施，构建了支撑稳增长调结构的制度体系。坚持周例会、月调度、季分析，建立完善“六本台账”，深入推进“四项对接”，持续开展“进千企、讲政策、解难题、促转型”活动，为重点企业解决融资550亿元、用工8万人、建设用地5万亩、减税16.5亿元。二是扩大有效需求。发挥投资关键作用，建立领导分包、观摩评价、集中开工等机制，省、市重点项目累计分别完成投资806亿元、2331亿元，固定资产投资年均增长15.6%，高于全省平均水平0.7个百分点。深入挖掘消费潜力，实施扩消费十大行动，社会消费品零售总额年均增长12.1%。积极扩大进出口，2017年外贸进出口总额达148.6亿元，居全省第2位。三是防范化解风险。积极化解企业风险，规模以上工业企业资产负债率38.8%，累计下降3.6个百分点。依法打击处置非法集资，有效维护了金融秩序和金融稳定。认真防范化解政府债务风险，严格规范举债行为，积极争取置换债券，加大土地出让等政府性基金收入，我市顺利退出债务预警地区。四是优化营商环境。深化“放管服”改革，加快建设国家级行政审批服务标准化试点市，深入推进并联审批、模拟审批、容缺审批，累计取消下放市直行政许可事项70项，压减市直行政职权1577项，取消中介服务事项88项，砍掉各类证明226项，行政审批办结时限全部压缩到法定时限的30%以内。新登记各类市场主体12.2万户，年均增长16%。

（二）调结构强供给，产业转型步伐加快。牢牢把握供给侧结构性改革这条主线，扎实推进“三去一降一补”，供给结构的适应性和灵活性不断提升。一是加快工业转型升级。全面推进传统产业转型，深入开展“三大改造”，以十大工业项目为龙头，带动实施转型升级项目495个，完成投资1093亿元。启动大企业集团培育工程，支持佰利联并购四川龙蟒，推动风神轮胎与意大利倍耐力成功整合，促成新开源收购美国BV生物科技，超百亿元、50亿元企业分别达到2家、12家。加快培育电子信息、生物医药、新材料、新能源等战略性新兴产业，占规模以上工业比重累计提高1.6个百分点。大力发展数字经济，与浪潮、华为实现战略合作，浪潮云计算中心、蛮蛮云一期建成投运，成功创建宽带中国示范城市。两化融合深入推进，国家级两化融合管理体系贯标试点企业、省级两化融合示范企业分别达到5家、56家。削减煤炭过剩产能217万吨。示范区成功创建国家新型工业化产业示范基地，全市产业集聚区固定资产投资、主营业务收入年均增长21.1%、16.6%，孟州市、武陟县产业集聚区2017年度全省考核综合排序分别居第8名、第11名。二是大力发展现代服务业。深入创建国家全域旅游示范市，加快培育休闲养生、疗养度假、体育健身等康养新业态，积极发展太极拳教育培训、服装器械、文化创意等产业，旅游综合收入年均增长13.4%，云台山荣获中国质量奖提名奖，中国焦作国际太极拳交流大赛获评中国体育旅游精品项目，全域旅游全息信息系统建设经验在全国推广，我市成为国家级旅游服务综合标准化示范市、国家智慧健康养老示范基地。建成电商产业园14个，入驻电商企业494家，电商交易额年均增长27.7%，获批中国（郑州）跨境电子商务综合试验区试点城市。金融业规模持续扩大，累计引进金融机构18家，金融业增加值年均增长12.3%，金融机构本外币存贷款余额年均分别增长12.1%、12.6%。商贸物流业快速发展，万达广场、王府井百货、鸿运国际商城开业运营，我市被确定为全省物流标准化示范市。全市服务业“两区”入驻企业871家、吸纳就业4.3万人，星级特色商业区数量达到5家。商品房库存去化周期稳定在合理区间，房地产市场保持平稳健康发展态势。三是推动现代农业提质增效。粮食总产稳定在40亿斤，夏粮单产保持全省第一、全国领先，获得中国优质小麦之都授牌，全国首家小麦博物馆开馆。建成高标准粮田169.2万亩，发展节水灌溉面积23.2万亩。优质小麦、优质花生面积分别增加23万亩、10万亩，优质林果、优质草畜规模持续扩大，成功创建“四大怀药”国家现代农业产业园。新型农业经营主体稳步发展，农民合作社、家庭农场总数分别达5690家、813家，2017年农业产业化集群销售收入达653亿元。

（三）激活力添动力，发展动能不断增强。深入推进改革开放创新，内外结合、联动发力，高质量发展的内生动力不断增强。一是以改革增动力。深化财政体制改革，建立新的市与县（市）区财政体制，调动了各方培植税源、发展经济的积极性。深化国企改革，全面推进“僵尸企业”处置，提前完成省属企业剥离办社会职能工作，荣获全省国企改革攻坚战先进市。深化医药卫生体制改革，“两延伸、三提升、一集中、三聚焦”“四医联动”的医改经验在全省推广，“探索困难群众大病补充保险的焦作样本”入选全国推进医改服务百姓健康十大新举措。全面实施商事制度改革，农村土地承包经营权确权登记颁证工作基本完成，国土、电力、交通等领域改革稳步推进。二是以开放借外力。成功举办“一赛一节”、豫商大会等重大活动，全市引进省外境内项目数量、合同利用省外境内资金年均分别增长11.8%、22.6%。与华夏幸福、中国中铁、德国莱茵金属等一批行业龙头实现战略合作。成为中德工业城市联盟首批会员，与加拿大桑德贝市缔结国际友好城市。河南德众保税物流中心封关运行，焦作进口肉类指定查验场、鹏海保税博览城一期投入运营。三是以创新强内力。积极融入郑洛新国家自主创新示范区，“一区多园”加快建设，焦作高新区升级为国家级高新区，河南理工大学科技园开园运营，5家单位享受自创区辐射区政策红利。在全省首家设立改革创新奖、企业创新奖，表彰创新创业领军人才（团队）两批次41个，发放扶持资金5650万元，首批人才公寓投入运营。完善科技贷款贴息制度，发放科技贷款1.5亿元。新增省级工程技术研究中心27家、省级院士工作站9家、高新技术企业48家、省创新龙头企业9家。获得授权发明专利1366件，年均增长34.7%。多氟多、健康元、河南理工大学的3项创新成果荣获国家科技进步二等奖，我市成为河南省首批科技综合改革试点。

（四）强基础促协调，城市转型扎实推进。着眼推动协调发展，统筹新型城镇化建设和乡村振兴战略实施，城乡、区域融合发展步伐加快。一是加强中心城区建设。深入开展“四城联创”，强力推进十大基础设施重点项目，实施中心城区改造提升行动，完成城市道路新改建项目95个，集中供热、燃气普及率分别达80.9%、95%，垃圾、污水处理率分别达97.5%、95%，入选全国第三批城市双修试点城市。二是实施百城建设提质工程。坚持以城促产、以产兴城、产城融合，实施提质项目498个、完成投资345亿元，武陟产业新城建设经验在全省交流推广。三是加快特色小镇和美丽乡村建设。陈家沟太极小镇、云台古镇等项目加快推进，温县赵堡镇入选第一批中国特色小镇。实施六大提升工程，农村人居环境明显改善，沁阳、修武、博爱、温县入选全省美丽乡村建设示范县（市），孟州莫沟村、博爱小底村等一批乡村成为全国全省美丽乡村示范村。

（五）重生态强攻坚，绿色发展成效显著。坚持环境保护和生态建设双向发力，美丽焦作建设取得新进展。一是强力开展环境攻坚。实施蓝天工程行动计划，重拳开展北山治理，集中整治“污染围城”，强化“六控”措施，有效破解施工扬尘治理、渣土车监管、环卫精细作业等难题，累计取缔整治“散乱污”企业2361家，拆改燃煤小锅炉1730台，完成“双替代”10.8万户，PM10、PM2.5浓度和空气质量优良天数实现“两降一升”。加强水生态保护，全面推行河长制，地表水主要指标达到国家要求。启动土壤污染防治攻坚，城乡面源污染防治加快推进。二是加强生态体系建设。“两口一路”绿化工程全面完成，建成公路绿色廊道526公里，完成森林资源培育80.4万亩，成功创建国家森林城市。建立空气质量生态补偿机制，制定生态环境损害责任追究办法，生态文明制度体系日益完善。三是推进绿色循环发展。实施绿色建筑提升行动，大力发展装配式建筑，商砼产业园、静脉产业园加快建设。成功入选北方地区冬季清洁取暖试点城市，获得中央财政三年15亿元奖励补助。加强与国家部委沟通对接，争取国家资源枯竭城市转型专项资金16.5亿元。

（六）惠民生增福祉，社会事业全面进步。加大民生投入，补齐民生短板，公共服务水平和均等化程度不断提高。一是深入开展脱贫攻坚。贯彻精准扶贫、精准脱贫基本方略，建立“四个一”工作机制，突出抓好产业扶贫、金融扶贫、健康扶贫、电商扶贫、旅游扶贫等重点任务，累计稳定脱贫7.67万人，66个贫困村退出贫困序列。创新实施“两定制兜底线”等健康扶贫模式，托住了困难群众因病致贫返贫的底线。二是积极扩大就业创业。开展就业创业春风行动等活动，推荐就业岗位45万个，职业技能培训27.4万人次。发放创业担保贷款28亿元，外出务工人员返乡下乡创业4.3万人，创办企业2.4万个。城镇新增就业39万人，新增农村劳动力转移就业15万人。三是持续加强社会保障。城乡居民养老保险参保率达到99.5%。实现城乡居民医保管理“六统一”，在全省率先实行城镇职工医保按比例报销上不封顶政策，成为全国跨省异地就医住院医疗费用直接结算市。建立困难残疾人生活补贴和重度残疾人护理补贴制度，发放补贴1.1亿元。在全国率先为进城务工农民建立住房公积金专户，“公积金管理六部法”成为全国推广样本。四是大力发展公共事业。新建改扩建幼儿园389所、中小学340所，实现国家义务教育发展基本均衡县（市）区全覆盖。教育质量明显提升，共有33名学生被清华北大录取，今年高考一本上线5190人，比2013年增长129%，获得全国职业院校技能大赛一等奖3个，成为河南省中小学创客教育试点市。市人民医院综合病房楼等项目建成投用，15分钟卫生服务圈基本形成。建成基层综合性文化服务中心1345个，“百姓文化超市”获评全国文化馆（站）优秀群众文化品牌，我市成为国家公共文化云推广试点地区。成功举办省十二届运动会暨首届全民健身大会，成为全省首批全民健身示范城市，第七次获评全国群众体育先进单位。购置绿色环保公交车556台，成为全省首批公交优先示范创建城市。五是全面加强社会治理。依法做好信访稳定工作，推广“三调对接、四级联动”矛盾纠纷调处化解机制，“334楼院协商治理模式”被评为全国社区治理十大创新成果。扎实开展双拥共建，连续第七次获评省级双拥模范城。加强普法宣传教育，荣获全国“六五”法治宣传教育先进城市称号。严格落实安全生产责任，全面加强食品药品安全监管，深化平安焦作建设，深入开展扫黑除恶专项斗争，公众安全感进入全省前三名。

2014年以来，我们主动接受人大及其常委会依法监督，积极支持政协参政议政，认真办理人大议案、代表建议和政协委员提案。加强民主法治建设，全面推进依法行政，不断强化审计监督。深入推进政府系统党风廉政建设，持续加强政府自身建设，扎实开展党的群众路线教育实践活动、“三严三实”专题教育、“两学一做”学习教育，坚决执行八项规定和省市意见，持续纠正和整治“四风”，“三公”经费累计下降50.1%。民族宗教工作创新推进，妇女儿童、残疾人、红十字、慈善等事业健康发展，外事侨务、统计调查、对台、档案、史志、气象、地震、人防、援疆等工作都取得了新的成绩。

特别是市第十一次党代会以来，在市委的正确领导下，着眼长远、科学谋划，长短结合、整体推进，市政府全力以赴抓了一批关系经济社会发展全局的大事要事，取得了一系列具有标志性意义的成就：一是开展解放思想活动，凝聚了发展共识。认真落实市委部署，连续三年先后开展了“近学许昌、远学扬州”“学深杭、促创新”“学成都佛山、促转型发展”等活动，对标先进、拉高标杆，树牢了新发展理念，坚定了战胜困难的信心，激发了奋力赶超的斗志，点燃了干事创业的激情，为推动高质量发展奠定了坚实的思想基础。二是推进郑焦深度融合，融入了全省大局。推动郑焦融合上升为省级战略，出台郑焦融合发展指导意见，积极构建“一枢纽、一新城、三平台、三基地”发展格局，确定南部产业新城、北部生态新城“南北呼应”区域整体开发思路，南北新城规划建设全面启动，郑焦综合交通走廊项目加快实施，为焦作发展打开了全新局面。三是加快实施“双十项目”，筑牢了发展支撑。坚持项目为王，强力实施十大工业项目，蒙牛乳业液态奶、河南超威正效一期、隆丰皮草自动制鞋园区等项目投产运行。扎实推进十大基础设施重点项目，焦作站南广场、南站房建成投用，苏蔺水厂一期通水，城市建成区黑臭水体基本消除，棚户区（城中村）改造全面推开，特别是弘扬南水北调焦作精神，众志成城、和谐征迁，仅用4个月时间，全面完成南水北调城区段绿化带征迁，创造了焦作城建史上的一个奇迹。四是深入开展“四城联创”，提升了城市品位。坚持“一城带四城、协同抓创建”，累计投入219亿元，强力实施背街小巷整治、农贸市场改造、违规广告拆除等工程，城市形象风貌、治理水平、文明程度明显提升，成功创建全国文明城市提名城市。五是聚焦发力北山治理，树牢了绿色理念。以壮士断腕的决心向多年痼疾宣战，严格执行北部山区生态环境保护条例，强力开展“两取缔一整顿一恢复”，非法采矿点和矿产品经营加工点全部拆除，完成矿山地质环境治理2.5万亩，北山生态修复治理成效明显，绿色发展理念深入人心。六是着力办好民生实事，增进了人民福祉。累计投入368.9亿元，全面推进重点民生实事和民生幸福工程，教育医疗、文化体育、困难群体帮扶、污染治理、社会保障等任务如期完成，人民群众的获得感幸福感显著增强。

各位代表，这些成绩的取得，是省委、省政府和市委正确领导的结果，是市人大、市政协、焦作军分区大力支持的结果，是全市人民团结拼搏的结果。在此，我代表市人民政府，向辛勤工作在各条战线的干部群众，向人大代表、政协委员，向各民主党派、工商联和无党派人士、各人民团体，向驻焦部队、武警官兵和公安干警，向离退休老领导、老同志，向关心支持焦作发展的社会各界人士，表示衷心的感谢并致以崇高的敬意！

各位代表，回顾过去的奋斗历程，总结取得的发展成绩，我们深切地感受到，做好新时代政府工作，必须把握好以下原则：一是坚持党对一切工作的领导。旗帜鲜明讲政治，树牢“四个意识”，做到“两个坚决维护”，认真执行党的路线方针政策。二是坚持稳中求进工作总基调。把“稳”和“进”有机统一起来，精准施策、精准发力，保持经济平稳健康运行。三是坚持新发展理念。坚定不移贯彻五大发展理念，加快构建现代化经济体系，推动经济社会高质量发展。四是坚持深化改革开放创新。用改革开放创新解难题、谋突破、促发展，激发市场活力和社会创造力，加快发展动能转换。五是坚持以人民为中心。牢记党的宗旨，始终把人民的利益作为一切工作的出发点和落脚点，用心用情用力在“七个有所”上下功夫，更好满足人民日益增长的美好生活需要。这些原则我们要继续坚持和发扬。

在总结成绩的同时，我们也清醒地认识到，经济社会发展还存在一些问题。一是经济下行压力依然较大，结构调整任务艰巨，“两高一资”产业比重较高，高新技术产业和服务业占比偏低，产品供给结构不优，破解结构性矛盾仍然任重道远。二是科技创新对产业转型、经济增长的驱动力还不够强，投入少、平台少、主体少的状况尚未得到根本性改变。三是中心城区首位度不高、承载力不强的问题仍需加快破解，城乡之间发展还不平衡。四是民生欠账依然较多，教育、医疗、文化、养老等公共服务体系需进一步完善，环保攻坚任务依然艰巨。五是政府职能有待进一步转变，有的政府工作人员能力素质不适应新时代要求，个别领域不正之风和腐败问题依然存在。我们要直面困难问题，采取有力措施，认真加以解决。

二、今后五年的目标任务

今后五年，是决胜全面建成小康社会、开启全面建设社会主义现代化新征程的关键时期。综合分析形势，深刻把握规律，焦作正处于转型攻坚、爬坡过坎的发展攻关期，又处在乘势而上、大有可为的战略机遇期。只要我们把握机遇、主动作为，持续发力、久久为功，就能破除传统路径依赖，重塑区域竞争优势，加快经济转型升级，实现跨越赶超、争先晋位，不断开拓焦作发展新境界。

今后五年的总体要求是：以习近平新时代中国特色社会主义思想为指导，深入贯彻落实党的十九大精神，坚持稳中求进工作总基调，坚持新发展理念，统筹推进“五位一体”总体布局和协调推进“四个全面”战略布局，坚持以供给侧结构性改革为主线，按照高质量发展要求，统筹稳增长、促改革、调结构、惠民生、防风险，发挥优势打好“四张牌”，坚决打好打赢“三大攻坚战”，提升“四城四区”建设水平，促进经济社会持续健康发展，决胜全面建成小康社会，建设全面体现新发展理念的示范城市，奋力谱写高质量发展的焦作篇章，在中原更加出彩中出重彩、更精彩。

根据市委十一届五次全会提出的奋斗目标，建议今后五年经济社会发展的主要目标是：加快建设“四个焦作”，跻身全省“第一方阵”，到2020年全面建成小康社会，并乘势而上全面推进新时代社会主义现代化建设，持续提升焦作在全国全省发展大局中的地位和作用，更好满足人民对美好生活的向往。

——发展质量效益显著提升。坚持高质量发展导向，产业结构、投资结构、环境质量、税收占比等质效指标持续优化，人均主要经济指标高于全国全省平均水平，社会文明程度、文化软实力显著提高，成为中原城市群战略支撑点和重要增长极。

——现代化经济体系更加完善。供给侧结构性改革取得明显进展，现代产业体系基本形成，科技创新体系全面构建，全要素生产率不断提高，产业结构迈向中高端，高新技术产业和战略性新兴产业占规模以上工业比重稳步提高。

——城乡建设实现重大突破。中心城区首位度明显提高，县城品质全面提升，常住人口城镇化率达到63%以上。乡村振兴取得重要进展，特色小镇和美丽乡村竞相发展，“城乡一体、四级联动”的新型城镇化发展格局基本形成。

——生态环境质量明显改善。环境空气质量持续好转，水环境质量不断提升，土壤环境质量逐步改善，城市绿地率和森林覆盖率明显提高。生态文明制度体系更加完善，生态产品供给持续增加，绿色低碳循环发展的能力不断增强。

——人民生活水平持续提高。居民收入与经济发展同步增长，教育、卫生、文化、体育等公共服务体系逐步健全，农村贫困人口全部稳定脱贫，社会就业更加充分，社会保障更加完善，治理体系更加科学。

实现上述目标，我们要围绕建设“一枢纽、一中心、两新城、两走廊、四绿带”发展格局，找准切入点、扭住发力点，推动区位优势、产业层次、基础能力、生态环境全面提升，为建设全面体现新发展理念的示范城市、在中原更加出彩中出重彩更精彩提供重要支撑。一是紧抓“一枢纽”，提升战略地位。围绕建设郑州大都市区门户枢纽城市、中原城市群和豫晋交界地区的区域性中心城市的发展定位，以交通枢纽设施建设为牵引点，强力推进高铁、高速、国道省道和跨黄河大桥等项目建设，整合工矿企业专用铁路、货站资源，以项目路线为“轴”，沿线两侧为“面”，以轴扩面、以轴带面，聚集人流、物流、信息流和资金流，推动沿线产业发展，形成高铁、铁路、高速、国道省道等枢纽经济叠加优势，以交通强枢纽、以枢纽聚产业，推动经济总量扩张、产业层次跃升，焦作在中原城市群核心区的战略地位更加突出。二是聚焦“一中心”，增强承载能力。大力推进中心城区建设，坚持整体规划、市场运作、连片开发、综合治理，统筹“四城联创”、城市双修、棚户区改造、百企退城等重点任务，推进城市功能区建设，加强城市治理与服务，提升城市形象和品位；加快建设新城区，改造提升老城区，强化居住与就业、功能与生态协调发展，做到以新补老、以老促新、有机融合，推动新老城区协调发展，全面提升中心城区首位度和综合竞争力。三是做强“两新城”，实现两极带动。加快南部产业新城建设，积极承接郑州大都市区等先进产业转移，加快发展高端装备、智能制造、金融服务、科技研发、文化创意等主导产业，打造郑焦深度融合的桥头堡。加快北部生态新城建设，强力推进循环经济、高端会展、文化休闲、健康养生、医养结合等项目，谋划建设多点对接、畅通便捷的“南北双城”综合交通体系，推动产业新城和生态新城“南北呼应”，促进先进制造业和现代服务业融合发展，加快形成南北新城双轮驱动、带动全局的发展格局。四是构建“两走廊”，促进区域联动。加强与洛阳、新乡、济源的产业对接，深化装备制造、化工、新能源、生物医药等产业合作，促进优势互补，拉长产业链条，实现集聚发展，着力构建新焦济洛工业走廊。以郑焦物流融合示范区、焦作跨境电商生态圈、武陟公铁物流港、日照港博爱无水港等为依托，向南对接郑州航空港经济综合实验区、河南自贸试验区和跨境电子商务综合试验区，向北对接山西国家资源型经济转型综合配套改革试验区，大力发展冷链物流、电商物流、多式联运、物流金融、保税物流等新业态，着力构建连接南北、贯通豫晋的郑焦晋物流走廊。五是打造“四绿带”，织密生态网络。加快整合云台山、青天河、神农山、青龙峡、峰林峡等南太行旅游资源，统筹实施生态系统保护、森林植被恢复、精品线路规划和旅游客源管理，推动景区景点横向联合、利益共享，努力打造集生态观光、休闲度假、健康养生、山地运动为一体的南太行生态旅游文体康养集聚带。加快连接武陟、温县、孟州黄河文化旅游资源，以建设沿黄产业带为支撑，以建设沿黄公路为纽带，以嘉应观、妙乐寺、陈家沟、韩园、莫沟等为重点，努力打造集文化休闲、生态观光、民俗体验为一体的黄河生态文化旅游带。加快南水北调绿化带建设，以城区段为重点，努力打造集旅游观光、生态景观、文化产业、休闲商业为一体的南水北调生态休闲带。加快大沙河生态项目建设，努力打造集生态体验、水景观赏、健身运动为一体的大沙河滨水景观带，让青山和绿水常在，让美丽与发展同行，全力建设宜居宜业生态文明城市。只要我们说了算、定了干，困难再大勇向前，美好的发展愿景就一定能早日实现。

三、下步重点工作

年初，市委十一届六次全会暨市委经济工作会议确定了全年经济社会发展主要目标（地区生产总值增长8%，在实际工作中争取更高质量和更好效益，规模以上工业增加值增长8.5%左右，第三产业增加值增长9.5%以上，全社会固定资产投资增长11%以上，社会消费品零售总额增长11.5%左右，进出口保持平稳增长，一般公共预算收入增长8.5%以上，城镇居民人均可支配收入增长8%，农村居民人均可支配收入增长8.5%），经过全市上下奋力拼搏，上半年主要指标实现“双过半”。下步，我们要坚定信心、强化措施，全面完成今年各项目标任务，全力推动经济社会高质量发展。重点做好以下工作：

（一）持续精准发力，坚决打好打赢三大攻坚战。提高政治站位，紧盯年度目标，强力开展攻坚，确保风险隐患有效控制，脱贫任务全面完成，环境质量持续改善。

防范化解重大风险。突出治乱象、抓规范、强监管，深入开展互联网金融专项整治、交易场所清理整顿，严厉打击非法集资等违法金融活动，加快政府融资平台市场化转型，坚决守住不发生区域性金融风险的底线。用好用活政府新增债券和置换债券等政策，防范化解政府债务风险，确保市本级专项债务率低于2017年度。发挥债权人委员会、还贷周转金等作用，着力化解企业联保互保、杠杆融资等风险隐患。

深入推进精准脱贫。持续实施打好精准脱贫攻坚战三年行动计划，确保1.24万贫困人口脱贫。加强贫困村饮水安全工程、电网改造升级、通村硬化路等基础设施建设，确保完成年度任务。谋划成立产业扶贫基金，坚持“一村一品”，大力发展特色产品加工、乡村旅游、农村电商等产业，再建成一批产业扶贫基地、扶贫车间。全面推广“两定制兜底线”健康扶贫模式，完善建档立卡贫困家庭学生教育资助制度，完成农村危房改造任务，做好农村低保提标工作，稳定实现“两线合一”，加快实现“两不愁三保障”。同时，做好结对帮扶工作，助力嵩县脱贫攻坚。

强力开展污染防治。积极调整产业结构、能源结构和交通结构，严控扬尘、燃煤、工业、机动车污染，加强应急管控，强化沿太行山污染传输通道区域综合治理，PM10、PM2.5年均浓度和优良天数达到省定标准。严格落实河长制，持续开展河流清洁、入河排污口整治、非法采砂和侵占水域岸线治理等专项行动，确保4个省控断面和18个市控断面稳定达标，确保通过全国水生态文明城市验收。加强土壤污染防治，深入开展土壤污染状况详查，推进土壤污染管控和修复试点示范工作。统筹山水林田湖草整体保护、系统修复、综合治理，加快实施国储林基地项目，完成森林资源培育18.1万亩。加快清洁取暖试点城市建设，完成“双替代”任务。实施绿色建筑提升行动，建成东部商砼产业园，加快建设静脉产业园、装配式建筑产业园、西部商砼产业园。

（二）深化供给侧结构性改革，加快构建现代产业体系。坚持质量第一、效益优先，持续提高供给质量，加快建设主导产业清晰、比较优势明显、集聚效应突出的产业体系。

大力扶持实体经济。全面落实国家、省减税降费政策，进一步完善我市产业、财税、土地等政策体系，切实减轻企业负担，筑牢高质量发展的实体经济根基。实施优化营商环境三年行动，深入开展“转变作风抓落实、优化环境促发展”活动，围绕企业开办、施工许可、要素供给、不动产登记、融资服务、法治保障等领域，全面压缩办理时限，提高政务服务效率，降低企业制度性交易成本。清理整顿机关事业单位所办企业，正确处理政府与市场、政府与企业的关系，充分发挥市场在资源配置中的决定性作用。贯彻落实全省促进非公有制经济健康发展大会精神，建立企业问题清单，研究制定政策措施，激活人才、技术、资本等关键要素，大力弘扬企业家精神，着力构建亲清新型政商关系，全面推动非公有制经济公平参与市场竞争，促进非公有制经济创新发展、转型发展、提质发展。

加快发展先进制造业。以高端化、绿色化、智能化、融合化、服务化为导向，大力调整产能、产业、产品结构，打造中原经济区高端制造业先进基地。深入开展“三大改造”。运用政策引导、环保倒逼等措施，加快实施87个绿色化改造项目，推动30家企业实施智能化改造、300家规模以上工业企业实施技术改造，确保技改投资占工业投资比重达到20%以上。持续化解过剩产能，全年退出煤炭过剩产能120万吨。积极培育大企业集团。加大项目建设、金融服务、人才引进等扶持力度，鼓励企业以补链、延链、强链为主线，开展并购联合和上下游整合，打造全产业链经济，推动重点培育对象做大做强。按照“个转企、小升规、规改股、股上市”的发展路径，再培育一批“专精特新”企业和“科技小巨人”企业。调整完善并强力推进十大工业项目。以十大工业项目为带动，全力推进210个重大工业项目，确保今年完成投资287亿元，力争中原内配国六发动机钢质活塞、龙蟒佰利联氯化法钛白粉、隆丰皮草综合加工产业园等项目年内建成投产，加快蒙牛百亿产业集群、多氟多新能源动力总成等项目建设。根据新变化，适时调整确定新的十大工业项目。实施百企退城行动。坚持搬迁一批、转型一批、淘汰一批，加快中心城区100家工业企业搬迁改造，年底前完成19家。大力发展“三新”经济。积极发展大数据、云计算、工业物联网、工业设计、数字经济等新经济新业态新模式，实施“千企上云”工程，加快推进焦作浪潮大数据产业园、蛮蛮云二期等项目，打造河南工业互联网重点综合平台和大数据产业基地。建设两化融合管理体系贯标企业30家、对标企业256家，推动300家企业“上云”。

推动服务业优化升级。加快生产性服务业向专业化和价值链高端延伸，生活性服务业向精细化和高品质转变，进一步扩大消费、拉动内需。促进旅游业转型发展。扎实做好国家全域旅游示范市创建工作，深入实施十大创新提升工程，强力推进云台古镇、陈家沟旅游服务综合体等重点项目，支持云台山等景区转型发展，加快建设国际知名文化旅游城市。促进文化康养业快速发展。办好中原康养产业（焦作）研讨活动，多渠道多形式引导社会资本进入健康养老产业，推进云阶康养小镇、南太行登山健身步道等项目建设，大力培育医疗旅游、休闲养生、疗养度假等康养新业态，加快建设中原经济区文化康养业引领基地。促进商贸物流业融合发展。深入推进内贸流通体制改革，加快建设省级物流标准化示范市，持续推动供应链创新与应用，积极推进武陟公铁物流港、日照港博爱无水港、中国宝武焦作现代综合物流园、万邦国际农产品冷链物流园、市物流标准化云平台等项目，依托鸿运国际商城、西部工业原料城，推进中心城区专业市场外迁。促进现代金融业创新发展。加大金融服务实体经济力度，推动政银企对接合作常态化制度化，完善焦作市智慧金融服务平台功能，积极推进市投资集团小额贷款公司设立工作，加强市县两级投融资平台对接，有效破解中小企业融资难、融资贵问题。完成县级农信社改制任务，加快组建焦作农商行。推进科瑞森、永威安防、云台山等企业上市工作，鼓励符合条件的优质企业发行企业债券，确保全市直接融资130亿元以上。促进电商产业做大做强。持续实施“双百”工程，加快建设焦作跨境电商生态圈。推广“电子商务+生产基地+社区直配”生鲜直销模式，推动社区电商与农村电商融合发展。加快县乡村三级电商服务站点建设，打通电商扶贫“最后一公里”。促进房地产业健康发展。规范房地产市场秩序，加快建立多主体供应、多渠道保障、租购并举的住房制度。

筑牢科学发展载体。深入推进示范区管理体制与人事薪酬制度改革，发挥国家级高新区政策优势，加快河南理工大学科技园、腾云电商产业园二期、科技总部新城等建设，全面打造智慧示范区。推动产业集聚区上档升级，抓好主导产业培育、专业服务园区建设、配套设施完善、园区招商运营等工作，力争主营业务收入增长10%以上。持续实施商务中心区提速、特色商业区提质工程，力争服务业“两区”社会消费品零售总额增长13%以上。

（三）全面推动改革开放创新，增强发展动力活力。坚持以改革释放生产力，以开放增强竞争力，以创新迸发创造力，为高质量发展提供强大动能。

统筹重点领域改革。着力破除体制机制障碍，推动重点领域和关键环节改革取得新突破。深化“放管服”改革。深入推进“一网通办”前提下的“最多跑一次”改革，完善审批服务事项目录，建立标准化工作规程，推进政务信息系统联通，年底前基本实现政务服务事项线上“一网通办”，线下“只进一扇门”，现场办理“最多跑一次”，完成好国家级行政审批服务标准化试点市创建任务。深化财政体制改革。加强新的市与县（市）区财政体制运行监测，加快预算管理制度改革，推进中期财政规划编制和财政项目库建设，实现预算绩效评价全覆盖。深化国有企业改革。全面完成市属企业公司制改革，做好驻焦央企“三供一业”分离移交，积极推进市直部门所属企业脱钩改制，抓好“僵尸企业”处置后续工作，确保顺利通过省政府验收。深化医药卫生体制改革。大力推进市域综合医改试点市建设，加快城市公立医院综合改革，实施基层医疗卫生服务体系三年提升计划，做实做好家庭医生签约服务，推行以按病种付费为主的多元复合式医保支付方式改革，健全医疗服务价格动态调整机制。同时，统筹推进教育、文化、国土、公共资源交易等领域改革。

提升开放发展水平。积极参加中博会等重大经贸活动，组织好我市在上海举办的项目推介活动，再引进一批与主导产业相配套的重点项目。积极融入“一带一路”，办好中德（焦作）高端制造企业合作论坛，加快推进中欧先进制造产业园建设。依托焦作海关、焦作进口肉类指定查验场等平台，争取纳入中国（河南）自由贸易试验区覆盖城市。积极申建德众综合保税区，支持博爱县、温县、修武县等产业集聚区申报省级经济技术开发区。

增强科技创新能力。积极融入郑洛新国家自创区空间布局，实施十大科技创新工程，加快建设科技综合体，打造科技创新“智谷”，推动郑洛新焦创新资源互通共享、优势互补、协同发展。突出企业创新主体地位，新增高新技术企业15家，总数突破100家。推进国家知识产权局专利审查协作河南中心尽快在焦作落地，新增省级工程技术研究中心10家、企业技术中心2家。加大财政科技投入，深化科技经费管理改革，建立科技企业投资引导基金，推动科技银行贷款体系全市全覆盖。深入实施青年人才工程，完善“1+6”引进培育人才（团队）政策，强力推进国际人才城、人力资源服务产业园、人才公寓等建设，为高质量发展提供强大智力支撑。

（四）加快新型城镇化建设，推动城乡区域协调发展。坚持以人的城镇化为核心，以提高城镇化质量为关键，推动城乡区域融合一体发展，加快建设中原经济区城乡协调发展示范基地。

全面推进郑焦深度融合。积极推进郑焦深度融合发展战略，落实郑州大都市区发展行动方案，主动对接、加强运作，尽快签订合作协议。加快新焦城际铁路、焦济洛城际铁路、呼南高铁豫西通道、焦平高速、焦唐高速、沿太行高速等项目前期工作，加快孟州黄河大桥等项目建设，深入开展詹店黄河大桥、东海大道南延黄河大桥、焦温快速通道南延黄河大桥等项目研究论证工作，打造郑焦深度融合的现代综合交通体系。推进南部产业新城、北部生态新城区域整体开发，启动黄沁河金三角地带和郑焦融合示范区规划工作。深化郑焦物流、文旅等领域合作，促进人才、科技、教育、医疗等资源共享，更好惠及两市人民。

加快中心城区改造提升。以“四城联创”为载体，实施中心城区改造建设提升工程，增强城市承载力和竞争力。适时调整并强力推进十大基础设施重点项目。一是全面提速南水北调绿化带建设，今冬明春完成绿化工程，加快安置房建设。二是强力推进李河、群英河、瓮涧河等城区河道综合整治，完成南水北调截洪沟工程。三是加快中原路建设，抓好道路穿越南水北调、山阳故城和城际铁路等节点工程；加快焦温快速通道（西环路）项目建设。四是严格按照施工计划，加快推进东海大道项目。五是强化项目协调服务，全力推进焦作至荥阳黄河大桥及连接线建设。六是确保苏蔺水厂全面通水，府城水厂完成50%工程量。七是全力保障太焦铁路顺利实施，依托高铁综合优势，谋划推进南水北调城区段高铁片区、武陟高铁片区、博爱高铁片区建设。八是加快推进大沙河生态治理，实施水景观改造提升，完成城区核心段地形整理、基础绿化等任务。九是加快推进新河商务区高压线路归并改造工程和新河生态治理项目。十是加大棚户区（城中村）改造力度，加快征迁安置进度，确保完成年度目标任务。增强城市承载能力。推进世行贷款绿色交通项目，完成人民路、站前路、塔南路等道路改造，启动城市绿道建设。大力开展厕所革命。实施城区电网优化提升工程。集中供热、燃气普及率分别达到85%、97%。推进沿街建筑风貌整治、黄土裸露治理、屋顶绿化等一批“城市双修”项目，实施地下综合管廊工程，加快海绵城市示范区建设。提升城市治理水平。深化城管执法体制改革，加强市容市貌、交通秩序等治理，着力构建权责明晰、服务优化、科学规范、高效有序的城市治理新机制。

扎实推进百城建设提质。强力补短板、强功能、增效能、提品质，加快推进总投资1409亿元的511个百城建设提质项目，确保全年完成投资300亿元。年底前打通、改造25%以上的“断头路”“卡脖路”，完成50%以上主次干道和30%以上背街小巷弱电入地，县城公共供水普及率达到85%，燃气普及率达到90%。

推动特色小镇和美丽乡村建设。突出产业、文化、旅游、社区“四位一体”，加快培育产业特色鲜明、生态环境优美、多种功能复合的特色小镇，力争每个县（市）区新培育1~2个特色小镇。深入开展“五清五化”，加快建设“四好农村路”，全面改善农村人居条件，再打造一批美丽乡村示范村。

（五）实施乡村振兴战略，推进农业农村现代化。准确把握乡村振兴战略的总要求，全面落实乡村振兴规划，推动农业农村优先发展，加快实现农业强、农村美、农民富。

大力发展现代农业。深化农业供给侧结构性改革，促进农业增效、农民增收。提升农业综合生产能力。推进高标准粮田建设，新增节水灌溉面积3万亩，持续提升农业机械化水平。积极调整农业产业结构。加大“四优四化”工作力度，巩固育种科技优势，增加优质小麦、优质花生种植面积，因地制宜发展林果产业，大力发展“两牛”优势产业。加快建设“四大怀药”国家现代农业产业园，加大特色农产品品牌培育力度。培育壮大“旅游+”“生态+”等农业新业态，促进农村一二三产业融合发展。加快推动农业产业化。健全粮食、农产品精深加工产业链条，推进宁夏上陵牧业现代化奶牛养殖牧场等23个农业产业化重点项目建设，确保农业产业化集群销售收入超700亿元。

全面深化农村改革。完善承包地“三权”分置制度，健全县乡两级土地流转服务体系。做好农村土地承包经营权确权登记颁证扫尾工作，建成市县两级确权登记数据库及管理信息系统。深化农村集体产权制度改革，加快农村集体资产清产核资工作，有序推进集体资产股份合作制改革，促进集体经济发展。扎实开展农村房屋不动产登记工作，完善农民闲置宅基地和闲置农房政策，深入开展“空心村”治理，促进资源要素合理流动和优化配置，增加农民财产性收入。

加强乡村有效治理。坚持自治、法治、德治相结合，深化拓展“四议两公开”工作法，完善乡村矛盾调解和便民服务机制，推进乡村治理体系和治理能力现代化。加大农村普法宣传教育力度，加强农村道德建设，制定完善村规民约，积极营造邻里友善、乡风和谐、向上向善、互帮互助的农村文明新风尚。

（六）着力保障改善民生，提升公共服务水平。坚持以人民为中心的发展思想，尽力而为、量力而行，办好重点民生实事，全力解决好人民群众最关心最直接最现实的利益问题。

大力扶持就业创业。实施全民技能振兴、就业惠民和创业富民工程，做好高校毕业生、退役士兵、城镇就业困难人员、去产能分流职工、农民工等重点群体就业工作，推荐就业岗位10万个以上，城镇新增就业6万人，新增农村劳动力转移就业1.7万人。

健全社会保障体系。深入实施全民参保计划，扎实创建全国社会保险标准化建设先行城市。做好医疗救助与基本医保、大病保险、大病补充保险政策的衔接，提升重特大疾病医疗救助水平。巩固社会救助和保障标准与物价上涨挂钩联动机制，加大困难职工帮扶救助力度，推进农村低保制度和扶贫开发政策有效衔接。

提升公共服务能力。优先发展教育事业，持续提升教育质量，实施第三期学前教育行动计划、高中阶段教育普及攻坚计划，深化义务教育集团化办学。合理配置教育资源，调整优化幼儿园、中小学规划布局，加快韩公教育园区初中部项目建设，推动市实验幼儿园新区分园、市十二中东部校区规划建设。开展职业教育“双元”培育改革试点工作，进一步深化产教融合、校企合作。支持河南理工大学内涵式发展，提升焦作大学、焦作师专办学层次。全面开展健康促进工作，加强重大疾病预防控制，加快市级区域医疗中心建设，推动县域医疗服务一体化发展。健全公共文化服务体系，统筹规划建设市老干部活动中心、文化馆、博物馆、美术馆、档案馆、残疾人康复中心、青少年宫等公共服务设施。加大文物保护力度。做好太极拳申遗工作，做大做强太极拳文化产业。实施全民健身计划，完善城市社区“10分钟体育健身圈”。

推进社会治理创新。深入开展社会矛盾纠纷调处，完善社区治理体系，完成“334”楼院协商治理模式推广工作。深入推进平安焦作建设，全面落实安全生产责任制，深化交通、建筑、危险化学品、消防、非煤矿山、食品药品、校园安全等重点行业领域专项整治，持续开展扫黑除恶专项斗争，健全完善立体化社会治安防控体系，确保社会大局和谐稳定。

同时，积极支持驻焦部队建设，深入开展双拥共建，巩固军政军民团结，推进军民融合深度发展。做好民族宗教工作，支持工会、共青团、妇联等人民团体工作。加强外事侨务、对台、统计调查、地震、气象、人防、档案、史志、援疆等工作。

四、加强政府自身建设

新时代要有新作为，新使命需要新担当。政府系统要深入开展“质量、效率、法治”落实年活动，加快转变政府职能，持续改进工作作风，全面提升履职水平，增强政府执行力和公信力，切实做到为人民服务、受人民监督、让人民满意。

（一）加强政治建设。树牢“四个意识”，坚定“四个自信”，做到“四个服从”，深入推进“两学一做”学习教育常态化制度化，认真开展“不忘初心、牢记使命”主题教育活动，坚决维护以习近平同志为核心的党中央权威和集中统一领导，在政治立场、政治方向、政治原则、政治道路上，始终同以习近平同志为核心的党中央保持高度一致。自觉用习近平新时代中国特色社会主义思想指导实践、推动工作，以实际行动确保党中央、国务院政令畅通，确保省委、省政府和市委各项决策部署落地生根。

（二）严格依法行政。深化政府系统机构改革，构建与新时代发展要求相适应的现代化政府治理体系。加快建设法治政府，坚持集体学法、法律顾问等制度，严格依法履行职责，规范公正文明执法，加强普法宣传教育，把政府活动全面纳入法治轨道，做到依法决策、依法行政、依法治理、依法担当。自觉接受人大法律监督、工作监督和政协民主监督，主动接受社会监督和舆论监督，认真听取人大代表、政协委员，民主党派、工商联、无党派人士和各人民团体意见，做到有权必有责、用权受监督。

（三）强化责任担当。政府系统领导干部要弘扬焦裕禄同志的“三股劲”，走出机关、深入基层，在一线推进三大攻坚战、产业转型、项目建设、招商引资、民生实事等中心工作，敢于担当、主动作为，以钉钉子精神解决发展难题、推动工作落实。完善容错纠错机制，坚持“三个区分开来”，为勇于创新、担当作为的干部撑腰鼓劲。加强政府诚信建设，健全守信激励和失信惩戒机制，以政务诚信带动社会诚信。

（四）坚持廉洁从政。严格执行中央八项规定及实施细则，坚决纠正形式主义、官僚主义新表现，驰而不息纠正“四风”。严格履行全面从严治党主体责任和“一岗双责”，强化审计和巡视巡察结果运用，加强工程建设、土地出让、政府采购、产权交易等重点领域监管，扎牢织密制度笼子，坚决惩治腐败行为，推进以案促改制度化常态化，大力营造风清气正、干事创业的良好环境。

各位代表！核心凝聚力量，思想指引方向。让我们更加紧密地团结在以习近平同志为核心的党中央周围，以习近平新时代中国特色社会主义思想为指导，撸起袖子加油干，扑下身子解困难，推动经济社会高质量发展，为加快建设“四个焦作”、早日跻身全省“第一方阵”，建设全面体现新发展理念的示范城市，奋力谱写高质量发展的焦作篇章，在中原更加出彩中出重彩、更精彩而努力奋斗！
